# Post-translational modifications to hemidesmosomes in human airway epithelial cells following diacetyl exposure

**DOI:** 10.1038/s41598-022-14019-x

**Published:** 2022-06-13

**Authors:** So-Young Kim, Matthew D. McGraw

**Affiliations:** 1 Division of Pulmonary Medicine, Department of Pediatrics, University of Rochester Medical Center, 601 Elmwood Avenue, Box 850, Rochester, NY 14642 USA; 2 Department of Environmental Medicine, University of Rochester Medical Center, Rochester, NY 14642 USA

**Keywords:** Preclinical research, Chemical modification, Intermediate filaments, Mechanisms of disease

## Abstract

Diacetyl (DA; 2,3-butanedione) is a highly reactive alpha (α)-diketone. Inhalation exposure to DA can cause significant airway epithelial cell injury, however, the mechanisms of toxicity remain poorly understood. The purpose of these experiments was to assess for changes in abundance and distribution of hemidesmosome-associated proteins following DA exposure that contribute to DA-induced epithelial toxicity. Human bronchial epithelial cells were grown in submerged cultures and exposed to three occupationally-relevant concentrations of DA (5.7, 8.6, or 11.4 mM) for 1 h. Following DA exposure, epithelial cells were cultured for 4 days to monitor for cell viability by MTT and WST-1 assays as well as for changes in cellular distribution and relative abundance of multiple hemidesmosome-associated proteins, including keratin 5 (KRT5), plectin (PLEC), integrin alpha 6 (ITGα6) and integrin beta 4 (ITGβ4). Significant toxicity developed in airway epithelial cells exposed to DA at concentrations ≥ 8.6 mM. DA exposure resulted in post-translational modifications to hemidesmosome-associated proteins with KRT5 crosslinking and ITGβ4 cleavage. Following DA exposure at 5.7 mM, these post-translational modifications to KRT5 resolved with time. Conversely, at DA concentrations ≥ 8.6 mM, modifications to KRT5 persisted in culture with decreased total abundance and perinuclear aggregation of hemidesmosome-associated proteins. Significant post-translational modifications to hemidesmosome-associated proteins develop in airway epithelial cells exposed to DA. At DA concentrations ≥ 8.6 mM, these hemidesmosome modifications persist in culture. Future work targeting hemidesmosome-associated protein modifications may prevent the development of lung disease following DA exposure.

## Introduction

Diacetyl (DA; 2,3-butanedione) is a chemical flavorant often added to food and drink for its buttery flavoring^1,2^. Chemically, DA is an alpha (α)-diketone with a low boiling point and high vapor pressure^2,3^. These properties make DA a highly volatile compound. When inhaled at occupationally relevant concentrations, DA exposure has been associated with the development of bronchiolitis obliterans (BO). BO is a debilitating, fibrotic lung disease of the small airways, and when associated with DA exposure, this lung disease has been coined ‘flavoring-related lung disease’^4,5^. However, investigations into the mechanisms of DA toxicity that contribute to flavoring-related lung disease induction remain in their infancy.

Multiple in vitro models have been developed previously for exploring the potential mechanisms of flavoring-related lung disease^6–11^. DA exposure to primary human airway epithelial cells causes extensive, non-reducible crosslinking of keratins (KRT), best seen with a shift in molecular weight^7,11^. Keratins are one of the most common cytoskeletal proteins of the airway epithelium^12,13^. Their structure provides important mechanical stability. Hence, abnormal crosslinking of these essential intermediate filaments likely contributes to epithelial instability. In addition to keratin crosslinking, other investigators have evaluated for transcriptomic changes in human airway epithelial cells exposed in culture to DA^14^. Consistent with prior proteomics data, pathways relevant to cytoskeletal structure were significantly modified in DA-exposed cultures. Collectively, these in vitro experiments support injury to the airway epithelium being a primary event in the initiation of flavoring-related lung disease.

One of the first cell types exposed to DA vapors is the airway epithelium. The airway epithelium consists of greater than 40 different cell types^15–17^. In simplest terms, airway epithelial cells are divided into two groups: luminal cells (LC’s) and basal cells (BC’s). LC’s are closest to the airway lumen and are in direct contact with the environment, while BC’s lie below the luminal layer and are adjacent to the basement membrane. LC’s are more commonly terminally differentiated while BC’s typically retain regenerative capacity with adherence to the adjacent basement membrane^16^. In steady state, BC’s are largely quiescent. Upon injury, BC’s migrate, proliferate, and differentiate for proper airway epithelial repair^18^. Regeneration of the airway epithelium is at least partly contingent upon basement membrane adherence for maintaining BC potential. KRT5 and KRT14 are two intermediate filaments commonly used to identify BC’s that also provide structural integrity for BC attachment to the basement membrane through hemidesmosomes^19^. In previous experiments, our group has shown marked damage to KRT5 after DA vapor exposure^11^. With time after DA exposure, damaged KRT5 proteins are degraded through the ubiquitin–proteasome pathway^11–13^. However, we have yet to characterize the downstream effects on hemidesmosomes following this initial KRT5 damage.

The purpose of the following experiments was to characterize how modifications to KRT5 affect hemidesmosomes in airway epithelial cells exposed to the flavoring chemical diacetyl (DA). Specifically, we evaluated changes in abundance and cellular localization of KRT5 in association with the hemidesmosome-associated proteins plectin (PLEC), integrins alpha 6 (ITGα6) and beta 4 (ITGβ4) at multiple time periods after DA exposure.

## Material and methods

### Chemicals

Diacetyl (DA; 2,3-butanedione) was purchased from Sigma-Aldrich (CAS no. 431-03-8, 99% pure; St. Louis, MO).

### Human bronchial epithelial cell lines and DA exposure

Human bronchial epithelial cells (16HBE 14o- and BEAS2B, Millipore, Burlington, MA) were cultured in Minimum Essential Medium Eagle (α-MEM, Sigma, St. Louis, MO) supplemented with 10% Fetal Bovine Serum (FBS, Corning, NY), 2 mM l-Glutamine and 1X Penicillin–Streptomycin solution (Thermo Fisher Scientific, Waltham, MA). Prior to culture, all flasks or plates were coated overnight with 34.5 µg/ml Collagen I (Corning, NY) in 70% Ethanol. All exposures occurred to cells in passage number 3–7. Authentication of cells for common airway epithelial genes and negative for mycoplasma were verified prior to exposure. Once cells reached approximately 70–80% confluence, submerged cultures were exposed to DA (5.7, 8.6, or 11.4 mM) by adding DA to the α-MEM culture media for 1 h. DA concentrations were extrapolated from previous experiments using in vitro exposure cultures^9,20^. The approximate vapor phase equivalent of DA for each concentration is 257 ppm, 387 ppm, and 514 ppm, respectively, using calculations published previously from a DA vapor cup exposure model^9,11^. Following exposure, cells were immediately rinsed with Dulbecco's phosphate-buffered saline (DPBS), and replenished with fresh medium. Cultures were then monitored for 1, 2, and 4 days after DA exposure. Cell viability was measured by MTT (see below) on Days 1, 2 and 4 after DA exposure and compared to media exposed culture conditions alone for percent change in absorbance. All DA exposures at each concentration were run in exposure replicates (3× each). For each time point after DA exposure, at least 4 culture wells were performed for protein collection and/or immunofluorescence as biologic replicates.

### MTT assay

To assess for cell death after exposure, MTT or 3-(4,5-dimethylthiazol-2-yl)-2,5-diphenyltetrazolium bromide (Sigma, St. Louis, MO) assay was measured at 1, 2, and 4 days after DA exposure. On the day of the assay, supernatant media was removed and replaced with 10 µl MTT (Sigma-Aldrich, St. Louis, MO) in 100 µl fresh culture medium, then incubated for 4 h at 37 °C and 5% CO_2_. Media from the MTT solution was removed carefully, and then 100 µl DMSO added to each well mixing thoroughly with the pipette. Sample absorbance at 570 nm and reference wavelength 690 nm was measured using a microplate reader SpectraMax M5 (Molecular Devices, San Jose, CA).

### WST-1 assay

To assess for cell death after exposure, water soluble tetrazolium-1 (WST-1, Sigma, St. Louis, MO) assay was performed at 1, 2, and 4 days after DA exposure. On the day of the assay, supernatant media was removed, replaced with 10 µl WST-1 (Sigma-Aldrich, St. Louis, MO) in 100 µl fresh culture medium, and then incubated for 2.5 h. at 37 °C and 5% CO_2_. Sample absorbance at 440 nm and reference wavelength 660 nm was measured using a microplate reader SpectraMax M5 (Molecular Devices, San Jose, CA).

### Immunocytochemistry staining

Airway epithelial cells (both 16HBE’s and BEAS2B’s) were grown on the collagen coated 15 mm coverslips (Chemglass Life Science, Vineland, NJ) and subjected to immunocytochemistry staining. At Days 1, 2 and 4 after DA exposure, cells were fixed for 10 min using 4% paraformaldehyde (PFA) and then permeabilized with 0.1% Triton-X-100 in PBS for 15 min. Fixed cells were then blocked with 2% bovine serum antigen (BSA) in phosphate-buffered solution (PBS) for 1 h and then incubated with the primary antibody of integrin beta 4 (ITGβ4, CD104; 1:250, Abcam, Cambridge, MA), Keratin 5 (KRT5; 1:250, Invitrogen, Waltham, MA), or integrin alpha 6 (ITGα6; 1:250, Biolegend, San Diego, CA) overnight at 4 °C. Cells were washed, incubated with the fluorescent secondary antibody (1:500–1000, Invitrogen, Waltham, MA) for 1 h, and mounted with DAPI Fluoromount medium (Southern Biotech, Birmingham, AL). Images were acquired using a fluorescence phase contrast microscope (Leica DM6000, Wetzlar, Germany) equipped with a digital camera (Hamamatsu orca-ER C4742-80, Japan). All image processing was performed using ImageJ program (NIH, Bethesda, Maryland).

### Western blotting

Samples were homogenized in RIPA lysis buffer (Thermo Scientific, Rockford, IL) supplemented with a protease inhibitor cocktail (Roche, Mannheim, Germany). Following centrifugation at 12,000 rpm for 20 min at 4 °C, soluble supernatant fractions were collected for total protein and western blot analysis. Total protein concentrations were determined by BCA assay kit (Thermo Scientific, Rockford, IL). Ten micrograms (μg) total protein were resolved in stain-free, pre-cast 4–15% gradient Tris–Glycine gel (Bio-Rad, Hercules, CA), and stained with primary antibodies of keratin 5 (KRT5; 1:2000, Biolegend, San Diego, CA), integrin beta 4 (ITGβ4, CD104; 1:1000, Invitrogen, Waltham, MA), and plectin (PLEC; 1:1000, ThermoFisher, Waltham, MA). Gels were transferred to 0.2 µm nitrocellulose membrane (Pall Corporation, NY). HRP and SuperSignal West Pico chemiluminescent substrates (Thermo Scientific, Rockford, IL) were used to detect protein signal intensity. Image Lab software (Bio-Rad, Hercules, CA) were used for target protein normalization and quantification. Full length blots were included in the online supplement.

### ELISA

Supernatant from 16HBE cell cultures were collected at various time points after DA exposures. The levels of matrix metalloproteinase-9 (MMP-9) in cell supernatant were determined using ELISA kits from R&D systems (Minneapolis, MN) according to manufacturers’ recommendations. One hundred (100) µl of supernatant from cell culture was used for MMP9 ELISA. MMP-9 activity was measured at the wavelength 450 nm using a microplate reader and reference wavelength of 570 nm.

### Statistics

Prior to analysis, all data were graphed using Prism 8.0 (GraphPad, La Jolla, CA) assessing for data distribution and variance within each exposure population. When normally distributed and with similar variance, a one- or two-way ANOVA was used with a p-value of 0.05 adjusted for multiple comparisons.

## Results

### Epithelial cell viability with respect to diacetyl (DA) concentration and time

**Figure 1:**
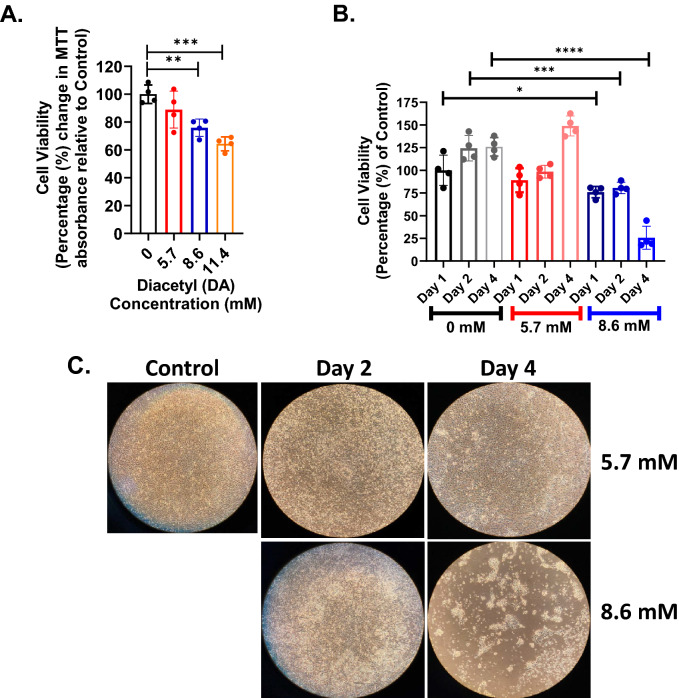
Cell viability with respect to concentration and time after DA exposure. (**A**) Cell viability decreased significantly at Day 1 in airway epithelial cultures exposed to DA concentrations of 8.6 mM and 11.4 mM, but not 5.7 mM DA, compared to PBS-exposed control cultures. MTT was measured within each culture well (n = 4/group) and calculated as a percentage change relative to non-exposed control wells. (**B**) Cell viability with respect to time after exposure (Days 1, 2, and 4) in non-exposed controls (black columns), 5.7 mM DA cultures (red columns), and 8.6 mM DA cultures (blue columns). Cell viability did not differ significantly in 5.7 mM DA-exposed cultures from PBS controls at any time point assessed. In 8.6 mM DA exposures, cell viability differed significantly at Days 1, 2 and 4 compared to controls (ANOVA with Dunnett’s correction, *p < 0.05, ***p < 0.001 and ****p < 0.0001). (**C**) Representative brightfield microscopy images of 16HBE cells at Day 2 and 4 after DA exposure. At Day 4, cell density improved following 5.7 mM DA exposure whereas 8.6 mM-exposed cells showed reduced cell density.

Cell viability decreased significantly in airway epithelial cultures exposed to DA at concentrations of 8.6 mM or 11.2 mM (Fig. 1A). At the lowest DA concentration (5.7 mM), cell viability did not differ significantly from control cultures. At later time points (Day 2 or 4 after DA exposure), cell viability remained significantly decreased compared to control samples exposed to 8.6 mM DA (Fig. 1B). Conversely, cell viability of cultures exposed to 5.7 mM DA did not differ significantly from controls at any of the evaluated time points following DA exposure. Cell viability was also assessed by water soluble tetrazolium-1 (WST-1) and was significantly reduced in epithelial cells exposed to 8.6 mM DA (Supplemental Fig. S1). Consistent with cell viability, brightfield microscopy images obtained at Days 2 and 4 post-exposure showed reduced cell density in cultures exposed to 8.6 mM DA in comparison to controls (Fig. 1C). Hence, airway epithelial cultures experienced significant toxicity at DA concentrations ≥ 8.6 mM.

### Diacetyl exposure induces keratin 5 crosslinking with cytoskeletal re-organization

**Figure 2:**
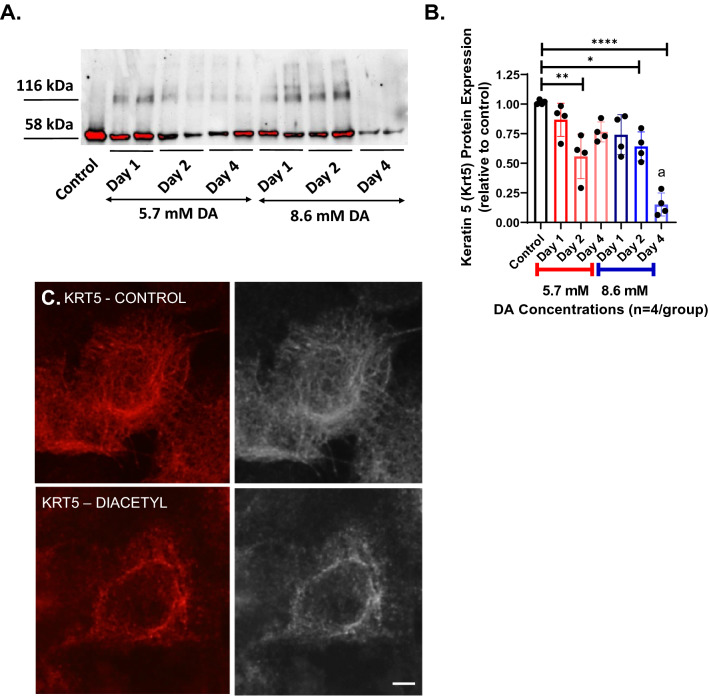
Diacetyl exposure induces keratin 5 crosslinking. (**A**) Representative western blot of keratin 5 (KRT5) from 16HBE culture homogenates at Days 1, 2 and 4 after DA exposure (5.7 and 8.6 mM). Known molecular weight of KRT5 is 58 kDa. Following DA exposure, KRT5 expression was also detected at 116 kDa. By Day 4 after DA exposure, higher molecular weight band dissipated. (**B**) Quantification of total lane KRT5 abundance normalized to lane total protein. Total KRT5 abundance decreased significantly at Day 2 in 5.7 mM and 8.6 mM DA-exposed cultures compared to controls (ANOVA; **p < 0.01 and *p < 0.05, respectively; n = 4/group). At Day 4, KRT5 abundance remained significantly lower than controls (ANOVA; ****p < 0.0001). (**C**) Representative immunocytochemistry images of KRT5 in control cells (top) and cells exposed to 8.6 mM DA (bottom) (scale bar: 10 μm). Images obtained at Day 2 after DA exposure. In control cells, the intermediate filaments stained by KRT5 extend toward the periphery. In DA-exposed cells, KRT5 intermediate filaments aggregate near the nucleus.

One proposed mechanism of DA-induced toxicity in airway epithelial cells is through abundant protein damage^7,11^. Keratin 5 (KRT5) is one of the most common cytoskeletal proteins of airway basal cells, the primary progenitor cell of the airway epithelium^18^. Hence, we evaluated for KRT5 abundance (Fig. 2A,B) and spatial distribution (Fig. 2C) in culture following DA exposure. KRT5 crosslinking developed in DA-exposed cultures independent of concentration. KRT5 crosslinking is best appreciated with a shift in molecular weight from 58 to 116 kDa via western blot (Fig. 2A). These higher molecular weight bands of KRT5 persisted for 2 days after DA exposure but were not seen at Day 4. The relative abundance of KRT5 at Day 4 was also significantly reduced in 8.6 mM DA exposed cultures compared to controls, but was not significantly different in 5.7 mM DA-exposed cultures from controls (Fig. 2B).

Next, we assessed for changes in the spatial distribution of KRT5 after DA exposure using immunocytochemistry (Fig. 2C and Supplemental Fig. S2). In control epithelial cultures, intermediate filaments of KRT5 extend out from the nucleus to the cell’s periphery. In contrast, in DA-exposed cultures, KRT5 aggregate in perinuclear distribution. Collectively, these findings suggest that DA exposure significantly modifies both total abundance and spatial distribution of KRT5 in airway epithelial cells.

### Diacetyl-induced keratin crosslinking modifies hemidesmosome abundance and distribution

**Figure 3:**
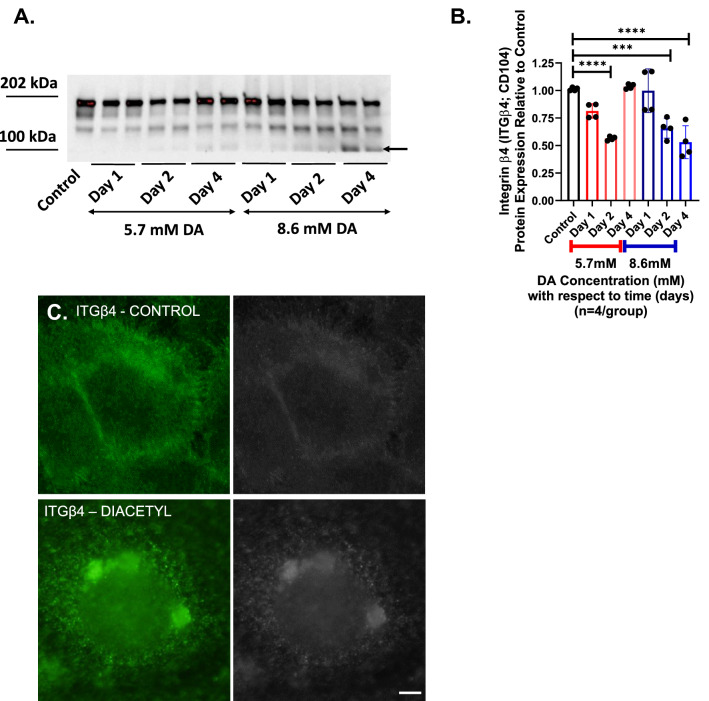
Modification to integrin beta 4 (ITGβ4) following DA exposure. (**A**) Representative western blot for integrin beta4 (ITGβ4) in epithelial cell homogenates in controls, Day 1, Day 2, and Day 4 after DA exposure. In cells exposed to 8.6 mM DA and cultured until Day 4, ITGβ4 expression at 202 kDa decreased with the appearance of ITGβ4 expression at approximately 100 kDa (far right two lanes, black arrows). (**B**) Semi-quantification of band density normalized for lane total protein. ITGβ4 abundance decreased significantly at Day 2 in both 5.7 mM and 8.6 mM DA at Day 2 (ANOVA, ****p < 0.0001 and ***p < 0.001, respectively). In 5.7 mM DA cultures, ITGβ4 abundance did not differ significantly compared to controls at Day 4 but remained significantly decreased relative to controls in 8.6 mM DA cultures (ANOVA, ****p < 0.0001). (**C**) Representative immunocytochemistry images of ITGβ4 in control cells (top) and cells exposed to 8.6 mM DA (bottom) (scale bar: 10 μm). Images obtained at Day 4 after DA exposure. In controls cells, ITGβ4 expression localizes primarily along the cell membrane. In DA-exposed cells, ITGβ4 expression localizes within the cytoplasm.

Keratins provide airway epithelial cells structural stability through the hemidesmosome-associated proteins plectin (PLEC), integrin beta 4 (ITGβ4) and integrin alpha 6 (ITGα6)^21^. Hence, we evaluated for changes in relative abundance and spatial distribution of these hemidesmosome-associated proteins after DA exposure. Similar to KRT5, ITGβ4 abundance decreased significantly by Day 2 in both 5.7 mM and 8.6 mM DA-exposed cultures compared to controls (Fig. 3A,B). In cultures exposed to 5.7 mM DA, total lane abundance of ITGβ4 did not differ from control cultures at Day 4. Conversely, ITGβ4 abundance in 8.6 mM DA-exposed cultures remained significantly decreased compared to controls at Day 4 (Fig. 3B). Additionally, a new lower molecular weight band via western blot appeared in 8.6 mM DA-exposed cells at Day 4 (black arrow, Fig. 3A). We further evaluated for this lower molecular weight band in ITGβ4 expression in 16HBE’s exposed to higher DA concentrations (Supplemental Fig. S3A) as well as in BEAS2B cells (Supplemental Fig. S3B). At both 11.4 mM and 22.8 mM DA concentrations in 16HBE’s, the expression of ITGβ4 was significantly reduced by Day 4 with the appearance of ITGβ4 expression at a lower molecular weight. Similar to 16HBE’s, this lower molecular weight band also appeared at Day 4 in BEAS2B cells exposed to DA.

By immunocytochemistry, spatial distribution of ITGβ4 in control cultures appeared primarily along the cell membrane connecting these cells to the basement membrane (Fig. 3C). In DA-exposed cultures, ITGβ4 expression was located primarily within the cytoplasm following both 5.7 mM and 8.6 mM DA exposures. Different from 5.7 mM DA exposure, the cytoplasmic location of ITGβ4 persisted in cultures exposed to 8.6 mM DA (Supplemental Fig. S4). Thus, DA exposure resulted in both a change in relative abundance as well as change in spatial distribution of ITGβ4, and at higher DA concentrations (≥ 8.6 mM), these changes in ITGβ4 distribution persist in culture.

**Figure 4:**
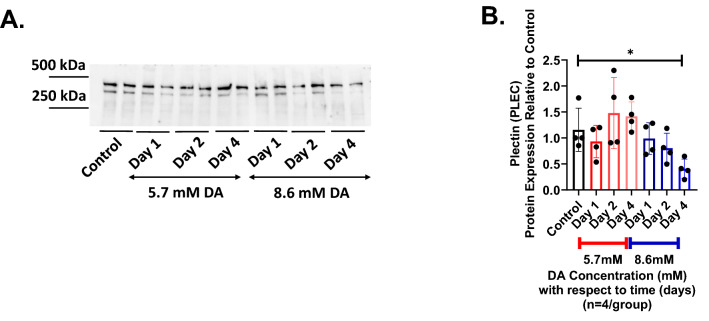
Changes in plectin abundance after DA exposure. (**A**) Representative western blot of plectin (PLEC) from epithelial cell culture homogenates at Days 1, 2 and 4 after DA exposure (5.7 and 8.6 mM). (**B**) Semi-quantification of PLEC abundance normalized to lane total protein that decreased significantly at Day 4 in cultures exposed to 8.6 mM DA compared to controls (ANOVA, *p < 0.05; n = 4/group).

Plectin (PLEC) is one of the hemidesmosome-associated proteins connecting KRT5 to the cytoplasmic tail of ITGβ4. No significant difference in PLEC abundance was seen at any of the time points assessed (Days 1, 2 or 4) with the lower DA exposure (5.7 mM) compared to control cultures (Fig. 4A,B). Conversely, PLEC abundance decreased significantly at Day 4 in cultures exposed to 8.6 mM DA compared to control cultures (Fig. 4B).

**Figure 5:**
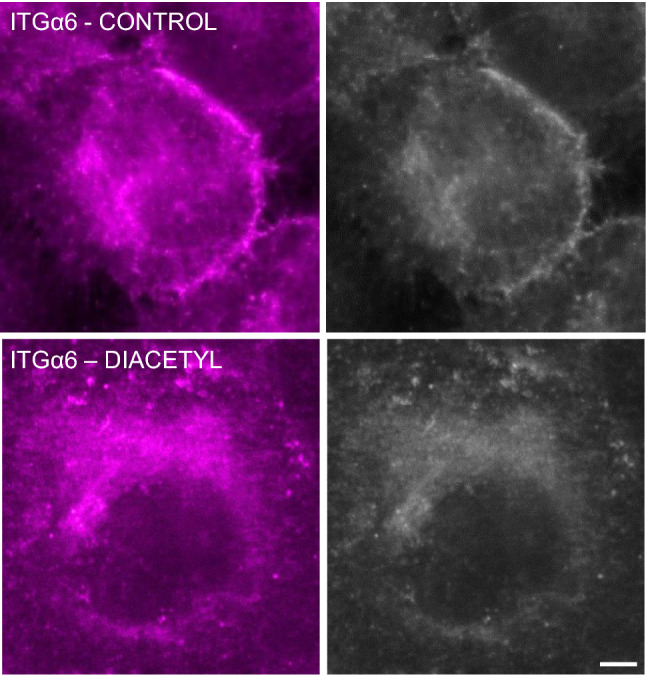
Integrin alpha 6 (ITGα6) distribution after DA exposure. Representative immunocytochemistry images of ITGα6 in control cells (top) and DA- exposed cells (bottom) at Day 4 (scale bar: 10 μm). In controls cells, ITGα6 expression localized primarily to the cell membrane. In DA-exposed cells, ITGα6 expression localizes primarily within the cytoplasm.

Integrin alpha 6 (ITGα6) interacts with ITGβ4 to form the heterodimer tail of the hemidesmosome, providing attachment to the basement membrane via laminin-332^19^. Similar to that of KRT5 and ITGβ4, the cellular distribution of ITGα6 changed significantly after DA exposure (Fig. 5 and Supplemental Fig. S5). Specifically, ITGα6 localized primarily to the cytoplasmic membrane in control cultures. Following DA exposure, ITGα6 distribution was seen throughout the cytoplasm and less along the cell membrane. Collectively, these changes in PLEC, ITGβ4 and ITGα6 support significant effects on hemidesmosome-associated proteins in human airway epithelial cells following DA exposure.

### Increased extracellular expression of MMP9 in DA-exposed cultures

**Figure 6:**
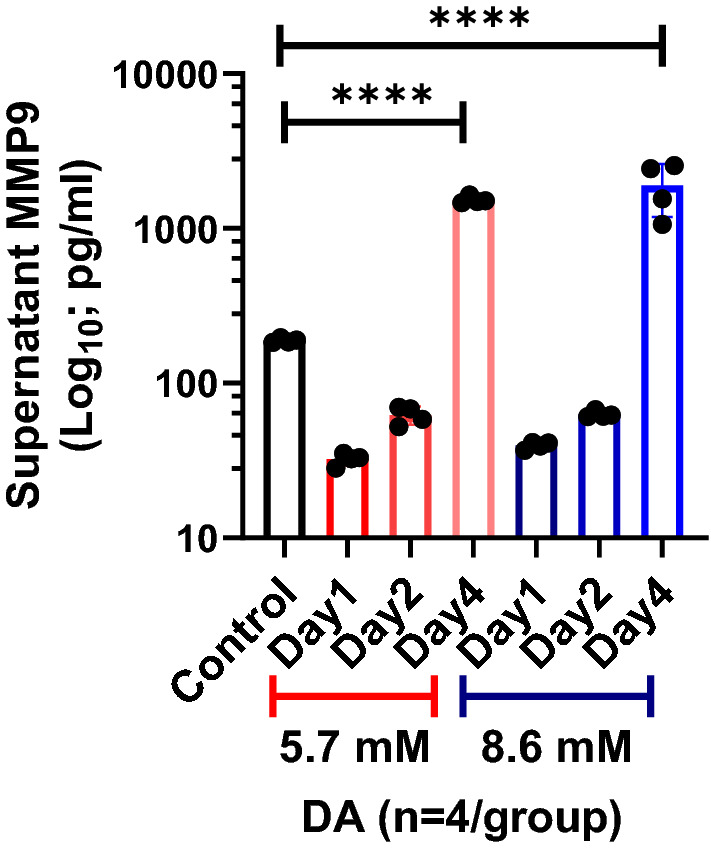
Expression of supernatant matrix metalloproteinase-9 (MMP9) in exposed epithelial cell culture. In DA-exposed cultures (5.7 mM or 8.6 mM), supernatant MMP9 expression increased significantly at Day 4 compared to control culture supernatants (ANOVA, ****p < 0.0001; n = 4/group).

Lastly, we measured supernatant matrix metalloproteinase-9 (MMP9) expression in exposed airway epithelial cultures, considering MMP9 has been shown previously to cleave the ectodomain of ITGβ4^22^. When evaluated temporally after DA exposure, supernatant MMP9 expression did not differ significantly from control cultures at Days 1 and 2 post-exposure with either 5.7 mM or 8.6 mM DA (Fig. 6). Conversely, MMP9 expression markedly increased at Day 4 in DA-exposed cultures compared to control supernatants. This increase in extracellular MMP9 associated temporally (at Day 4) with cleavage of ITGβ4 in DA-exposed cultures via western blot.

## Discussion

Diacetyl (DA) exposure to airway epithelial cells resulted in keratin 5 (KRT5) damage and significant downstream effects on hemidesmosome-associated proteins. The initial event triggered by DA exposure was KRT5 crosslinking. With sufficient time after DA exposure or at lower DA concentration, airway epithelial cells re-establish homeostasis through changes in abundance and distribution of keratin 5, plectin (PLEC), integrins alpha 6 (ITGα6) and beta 4 (ITGβ4). Conversely, airway epithelial cells exposed to higher DA concentrations develop reduced abundance of KRT5, PLEC, ITGα6 and ITGβ4 with perinuclear aggregation of these hemidesmosome proteins that associate temporally with increased expression of extracellular MMP9. Collectively, DA exposure results in, not only structural damage to important cytoskeletal proteins such as KRT5, but also modification to hemidesmosomes.

Hemidesmosomes (HD’s) are highly complex structures common to epithelial cells. These structures play an essential role in maintaining epithelial integrity through the stable adhesion of epithelial cells to the adjacent basement membrane^21^. Hemidesmosomes are grouped into two types: Type I and II HD’s^23^. Type I HD’s are common to pseudostratified epithelia and contain the 5 proteins of integrins α6β4, plectin isoform 1a (P1a), tetraspanin (CD151), bullous pemphigoid antigen (BPAG) 1 isoform e (BPAG1e, or BP240) and BPAG2 (also known as BP180 or type XVII collagen). Type II HD’s are seen in simple, non-stratified epithelia and contain all of the same proteins except for the two BP antigens. The intracellular binding partners for HD’s are the intermediate filaments KRT5 and KRT14 while the heterodimer pair of integrins α6β4 bind extracellularly to the adjacent basement membrane through laminin-322^21,23^.

Epidermolysis bullosa (EB) is a heterogeneous group of inherited or acquired blistering disorders whose common theme is dysfunctional or instable HD proteins^23–25^. Multiple epidermal layers, including but not limited that of the skin, nails, genitourinary and respiratory tracts, are often affected by the disorder. Patients manifest with epidermal fragility and recurrent blisters. EB is divided into three categories: EB simplex (EBS), junctional EB (JEB), and dystrophic EB (DEB)^25^. EBS is the most common type of EB, with dominant mutations seen in genes encoding the intermediate filament genes KRT5 and KRT14^23^. Mutations in KRT5 or KRT14 result in abnormal assembly and network formation of these essential intermediate filaments. These dysfunctional cytoskeletal proteins aggregate around the cell’s nucleus, a hallmark feature of EBS histologically^12,23^. This dysfunctional network of keratin proteins results in a cascade of downstream effects to the HD structure, ultimately resulting in hemidesmosome instability.

Following DA exposure, damage to the intermediate filament KRT5 occurs through non-enzymatic adduction of DA with arginine residues^26,27^. More specifically, DA functions as an electrophile that donates a bond to nearby nucleophiles within the cell^26^. This adduction can occur between two nearby keratins that are rich with arginine, resulting in crosslinking of KRT5. Previous investigations have shown that in primary human airway epithelial cells, other intermediate filaments such as KRT6 and KRT14, are also susceptible to this reaction^7,11^.

Here, we show KRT5 crosslinking triggers a cascade of events with changes in abundance and distribution of the hemidesmosome-associated proteins, including PLEC, ITGα6, and ITGβ4. At lower DA concentrations, the spatial distribution of KRT5, ITGα6, and ITGβ4 recovers with sufficient time after exposure. At higher concentrations, DA exposure resulted in persistent perinuclear aggregation of KRT5 and cytoplasmic localization of ITGβ4 and ITGα6. Additionally, modification to ITGβ4 occurred with reduced total ITGβ4 abundance. Potential mechanisms that would explain both reduced abundance and modification to ITGβ4 include: (1) ectodermal cleavage by matrix metalloprotease-9 (MMP9)^22^, (2) internal cleavage by caspase-3 and -7^28^, or (3) calcium-dependent calpain cleavage^29^. Following DA exposure, supernatant MMP expression increased significantly at Day 4 and associated temporally with the modifications seen to ITGβ4. Previous authors have shown activation of MMP9 in cultured corneal and epidermal keratinocytes at sites of epithelial erosion, and with inhibition of MMP9, epithelial erosions were reduced with reduced cleavage of ITGβ4^22^. Thus, cleavage of the ITGβ4’s ectodomain by MMP9 activation may be one potential mechanism to explain the late effects seen in ITGβ4 after higher concentration DA exposure. However, future experiments are required to validate whether MMP9 is the primary pathway contributory to ITGβ4 cleavage.

The current studies extend our mechanistic understanding of DA exposure on airway epithelial cells to the downstream effects on hemidesmosome-associated proteins. Considering hemidesmosome-associated proteins provide essential structural integrity to the airway epithelial cells, future therapies targeting hemidesmosomes may prevent both the short-term toxicity or long-term fibrotic lung disease associated with DA inhalation exposures.

## Supplementary Information

Supplementary Legends.

Supplementary Figures.

## Data Availability

The datasets used and/or analyzed during the current study available from the corresponding author on reasonable request.
