# Supplementary material for: Post-translational modifications to hemidesmosomes in human airway epithelial cells following diacetyl exposure
Source: Sci Rep. 2022 Jun 13;12:9738. doi: 10.1038/s41598-022-14019-x (PMC9192738; doi:10.1038/s41598-022-14019-x)
Supplement: Supplementary file 1 — Supplementary Legends. [file 41598_2022_14019_MOESM1_ESM.docx]

**SUPPLEMENTAL FIGURE LEGENDS**

**Figure S1.** **DA exposure decreases cell viability with respect to DA concentration and time.** (A) Cell viability measured via WST-1 (n=4/group) and percentage change calculated relative to control in cultures exposed to 5.7 mM and 8.6 mM at Day 1, 2 and 4 after DA exposure. Cell viability differed significantly in DA-exposed cultures compared to PBS controls with respect to time (ANOVA with Dunnett’s, ****p<0.0001). (B) Cell viability measured by MTT (n=4/group) in BEAS2B cells and calculated percentage change relative to control. Cell viability differed significantly in DA-exposed cultures compared to PBS controls with respect to time (ANOVA with Dunnett’s, ****p<0.0001).

**Figure S2. Keratin 5 (KRT5) aggregates in DA-exposed epithelial cells at Day 2 after exposure.** (A) Representative immunocytochemistry images of KRT5 in control cells (top) and cells exposed to 8.6 mM DA (bottom) (scale bar: 50 μm). Images obtained at Day 2 after DA exposure. In control cells, the intermediate filaments stained by KRT5 extend toward the periphery. In DA-exposed cells, KRT5 intermediate filaments aggregate near the nucleus.

**Figure S3**. **Integrin beta 4 (ITGβ4) expression after DA exposure in 16HBE’s and BEAS2B cells**. (A) Representative western blots of for ITGβ4 abundance in 16HBE cell homogenates following exposure to higher concentrations of DA (11.4 mM or 22.8 mM) with respect to time (Days 1, 2 and 4 after exposure). Exposure to either 11.4 mM or 22.8 mM DA resulted in reduced total ITGβ4 (202 kDa) and the appearance of multiple lower molecular weight bands at Days 1, 2 and 4 post-exposure. (B) Representative western blots of BEAS2B airway epithelial cell homogenates stained for ITGβ4 following exposure to 8.6 mM DA with respect to time (Days 1, 2 and 4 after DA exposure). Appearance of similar lower molecular weight band at approximately 100 kDa at Day 2 post exposure in BEAS2B cells.

**Figure S4. Cytoplasmic localization of integrin beta 4 (ITGβ4) in DA-exposed epithelial cells.** (A) Representative immunocytochemistry images of ITGβ4 in control cells (top) and cells exposed to 8.6 mM DA (bottom) (scale bar: 50 μm). Images obtained at Day 4 after DA exposure. In control cells, ITGβ4 localize to the cell membrane. In DA-exposed cells, ITGβ4 localize primarily to the cytoplasm.

**Figure S5. Cytoplasmic localization of integrin alpha 6 (ITGα6) in DA-exposed epithelial cells.** (A) Representative immunocytochemistry images of ITGα6 in control cells (top) and cells exposed to 8.6 mM DA (bottom) (scale bar: 50 μm). Images obtained at Day 4 after DA exposure. In control cells, ITGα6 localize to the cell membrane. In DA-exposed cells, ITGα6 localize primarily to the cytoplasm.
