# Supplementary material for: Post-translational modifications to hemidesmosomes in human airway epithelial cells following diacetyl exposure
Source: Sci Rep. 2022 Jun 13;12:9738. doi: 10.1038/s41598-022-14019-x (PMC9192738; doi:10.1038/s41598-022-14019-x)

Post-Translational Modifications to Hemidesmosomes in Human Airway Epithelial Cells Following Diacetyl Exposure

So-Young Kim, MS [1,2] and Matthew D. McGraw, MD[1,2]
[1]Department of Pediatrics, Division of Pulmonary Medicine and [2]Department of Environmental Medicine, University of Rochester Medical Center, Rochester, NY 14642

Supplemental Material

Figure S1.

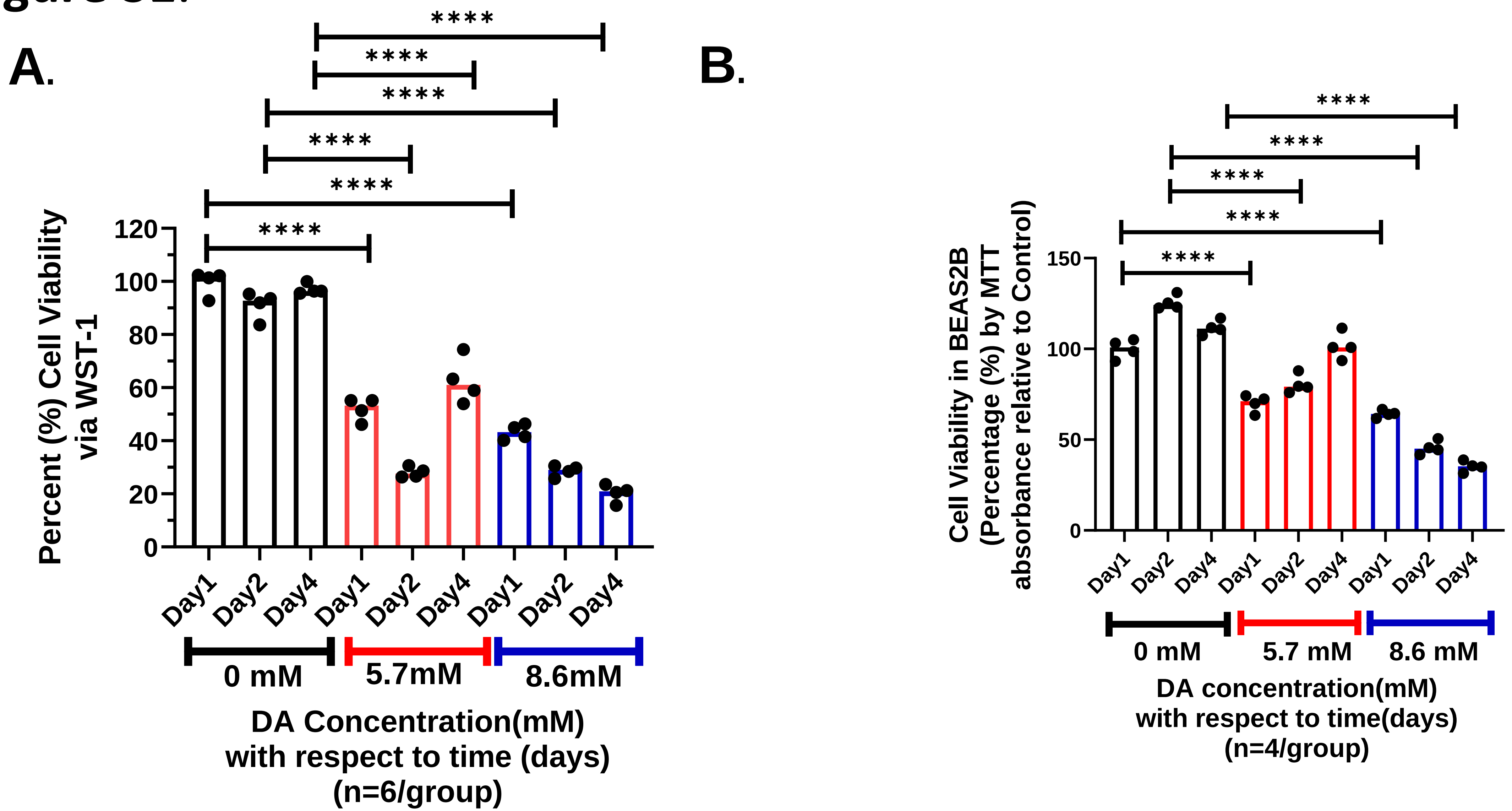

Figure S2.

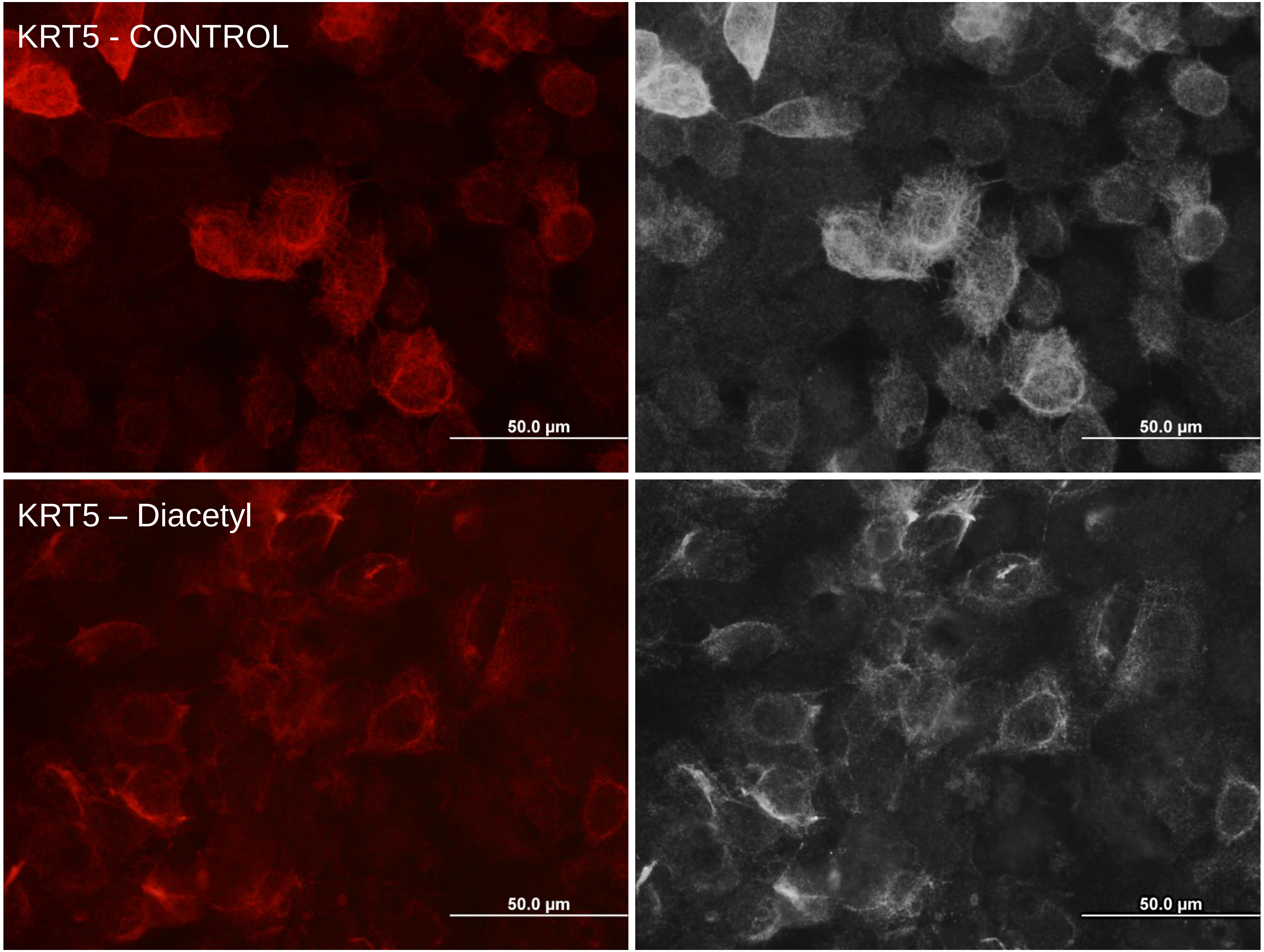

Figure S3.

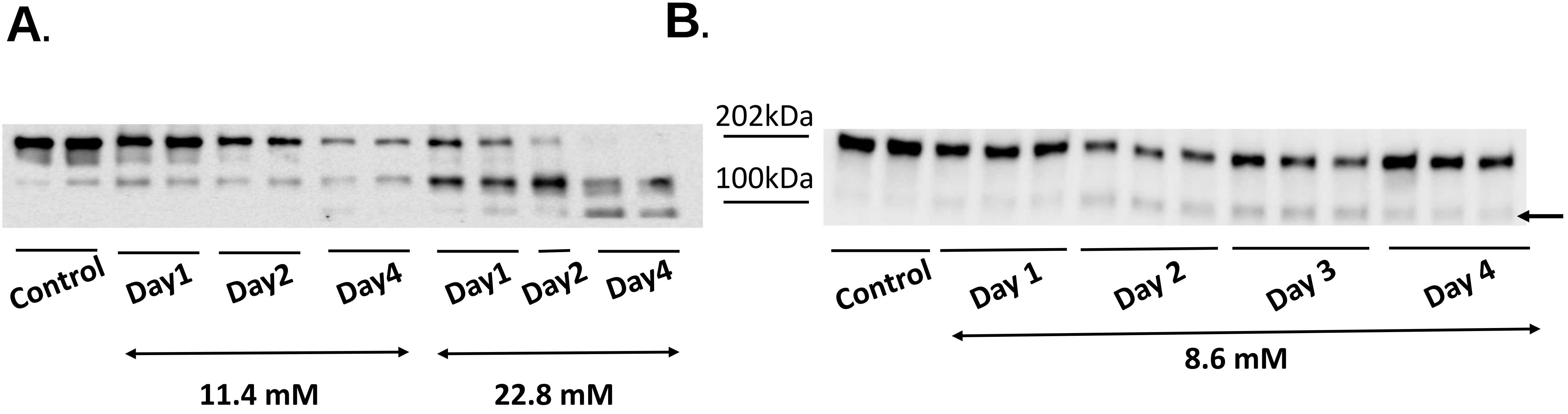
A.
B.
202kDa
100kDa
Control
Day1
Day2
Day4
Day1
Day2
Day4
11.4 mM
22.8 mM
Control
Day 1
Day 2
Day 3
Day 4
8.6 mM

Figure S4.

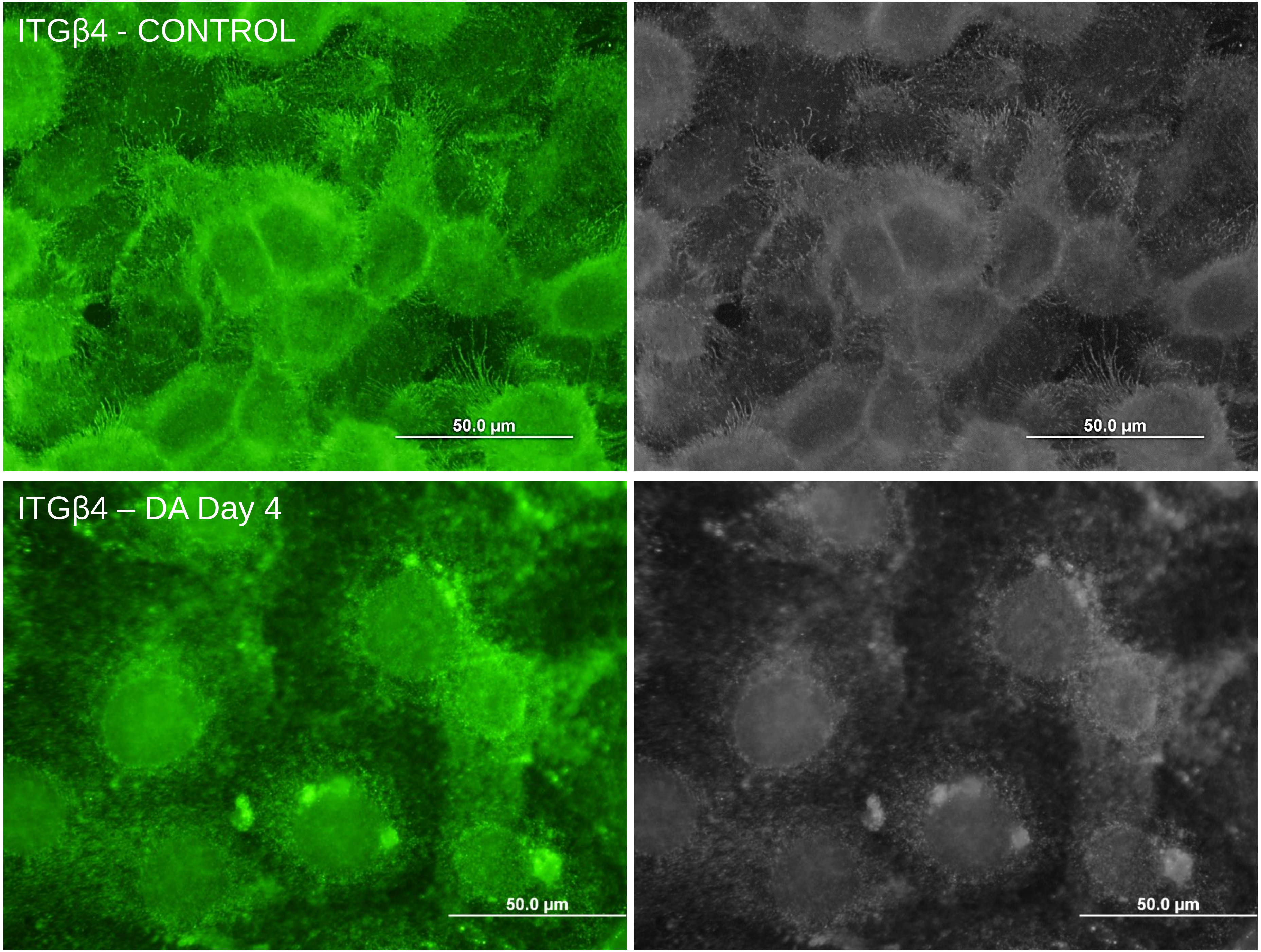

Figure S5.

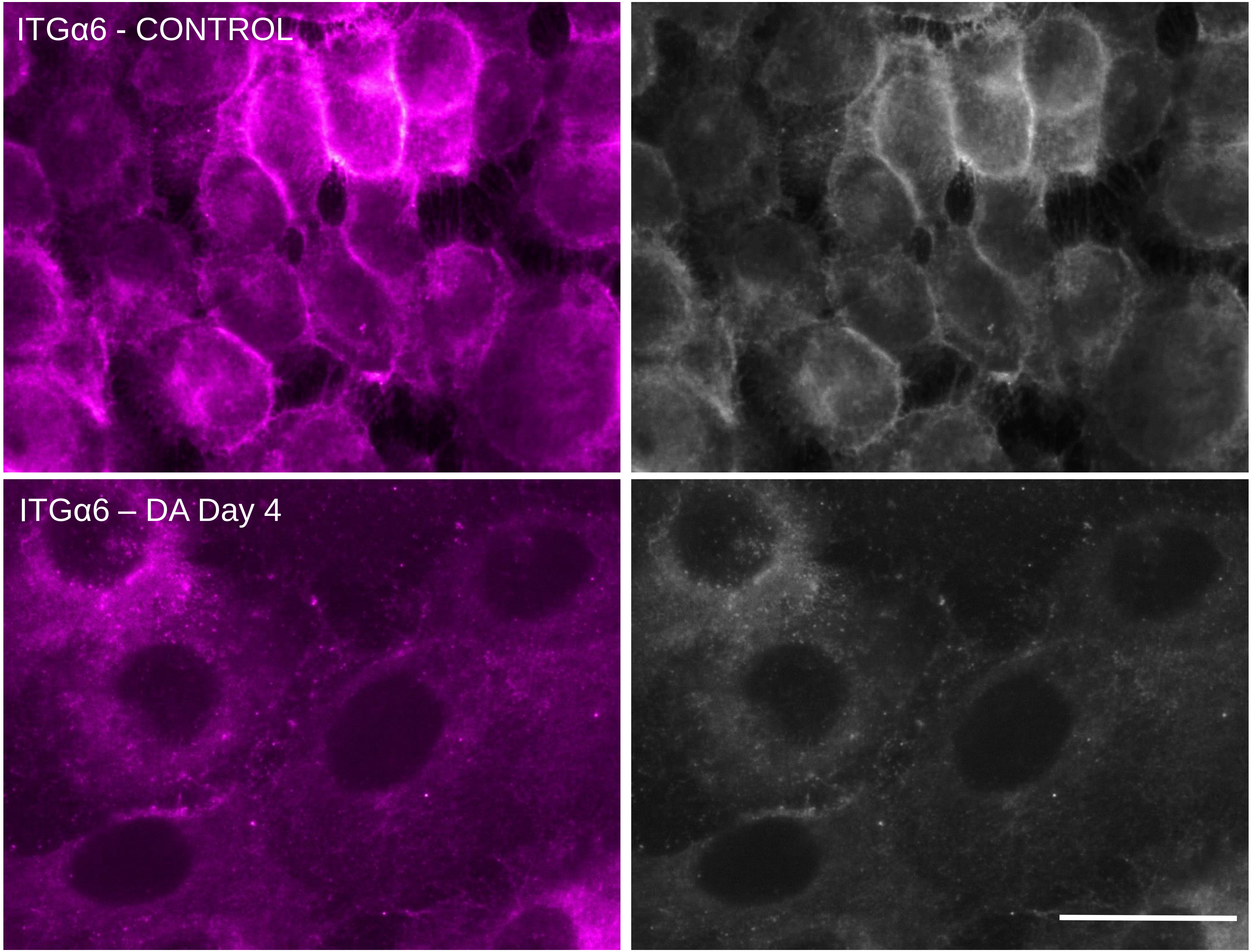

UNCROPPED BLOTS

Figure 2.

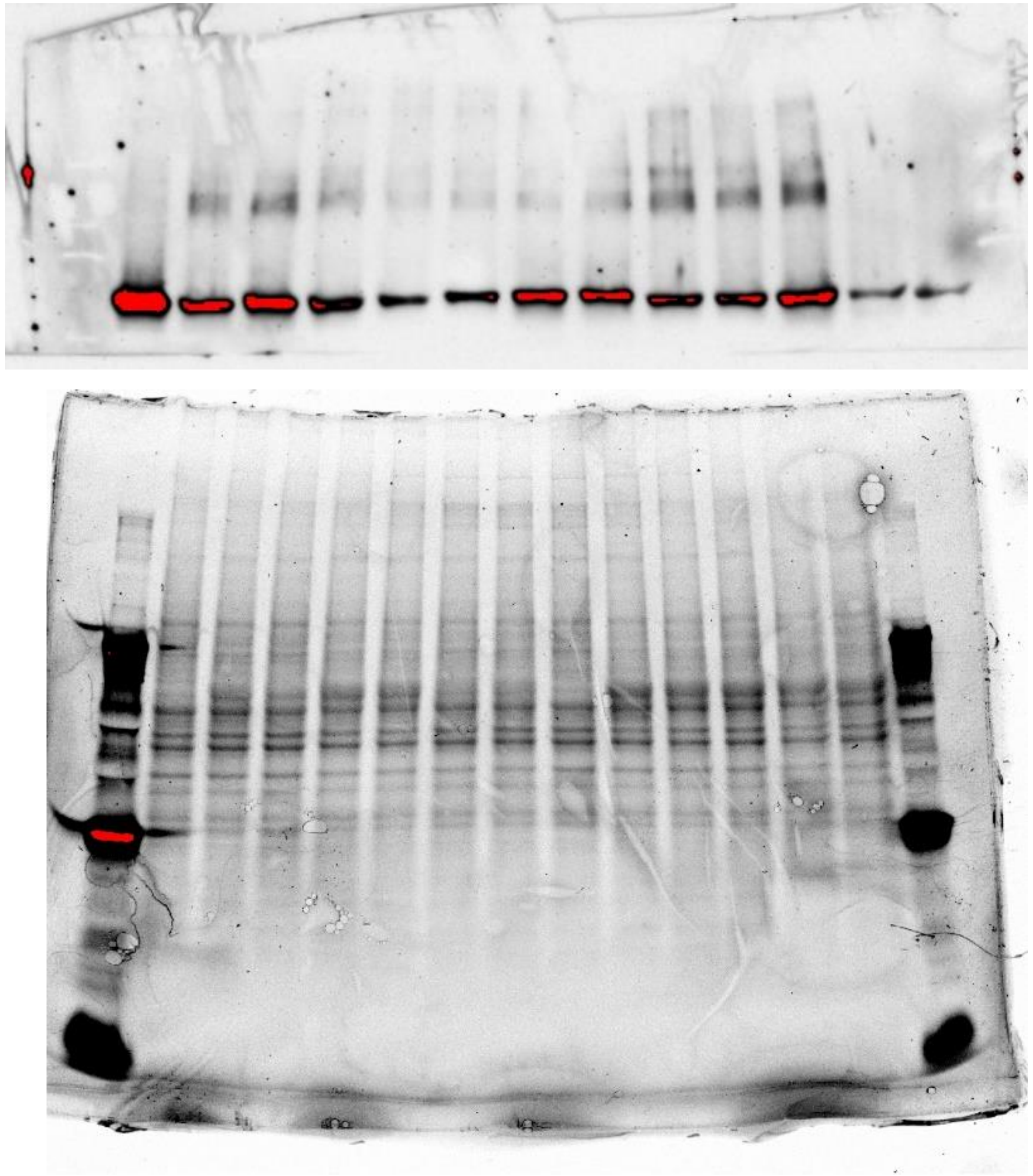

Figure 3.

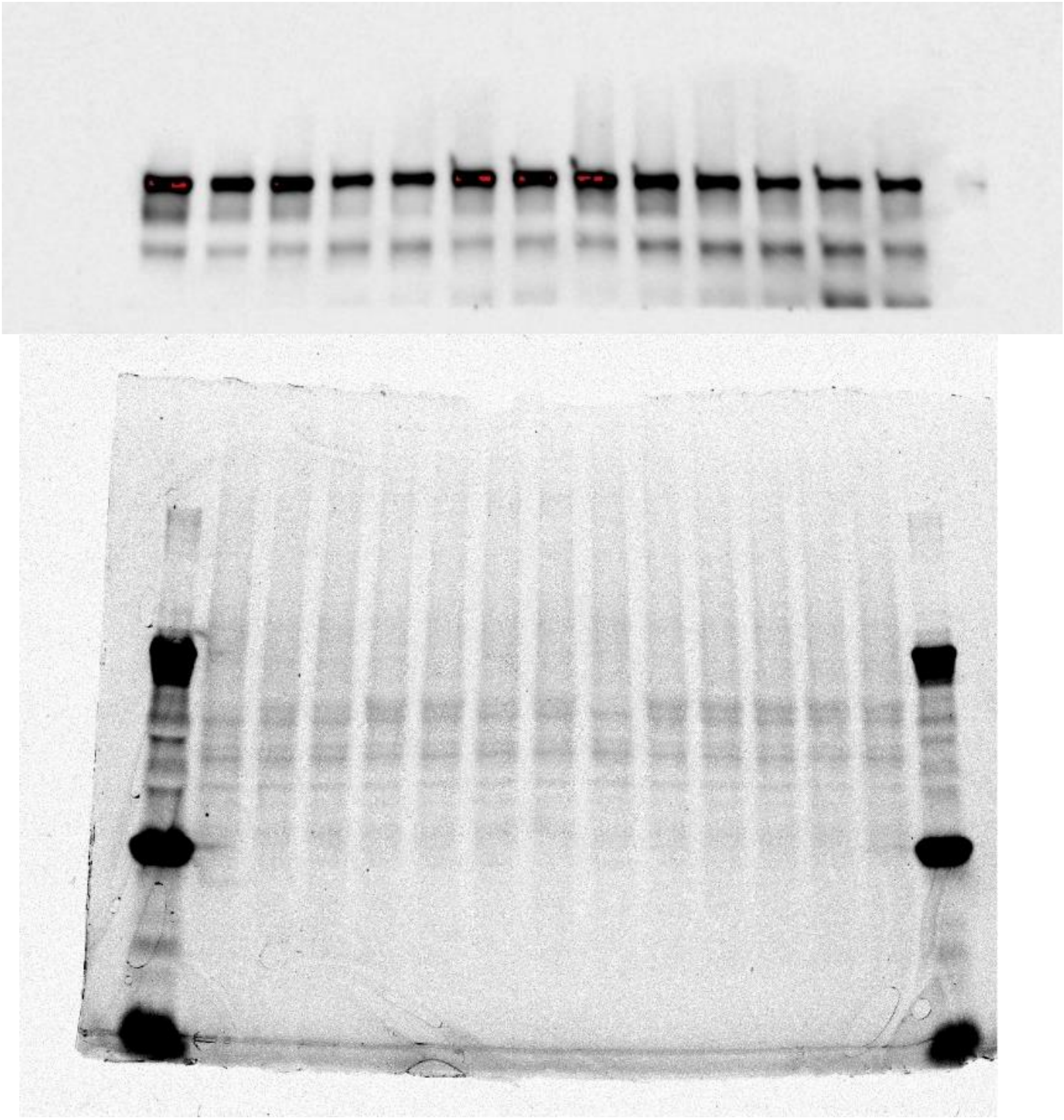

Figure 4.

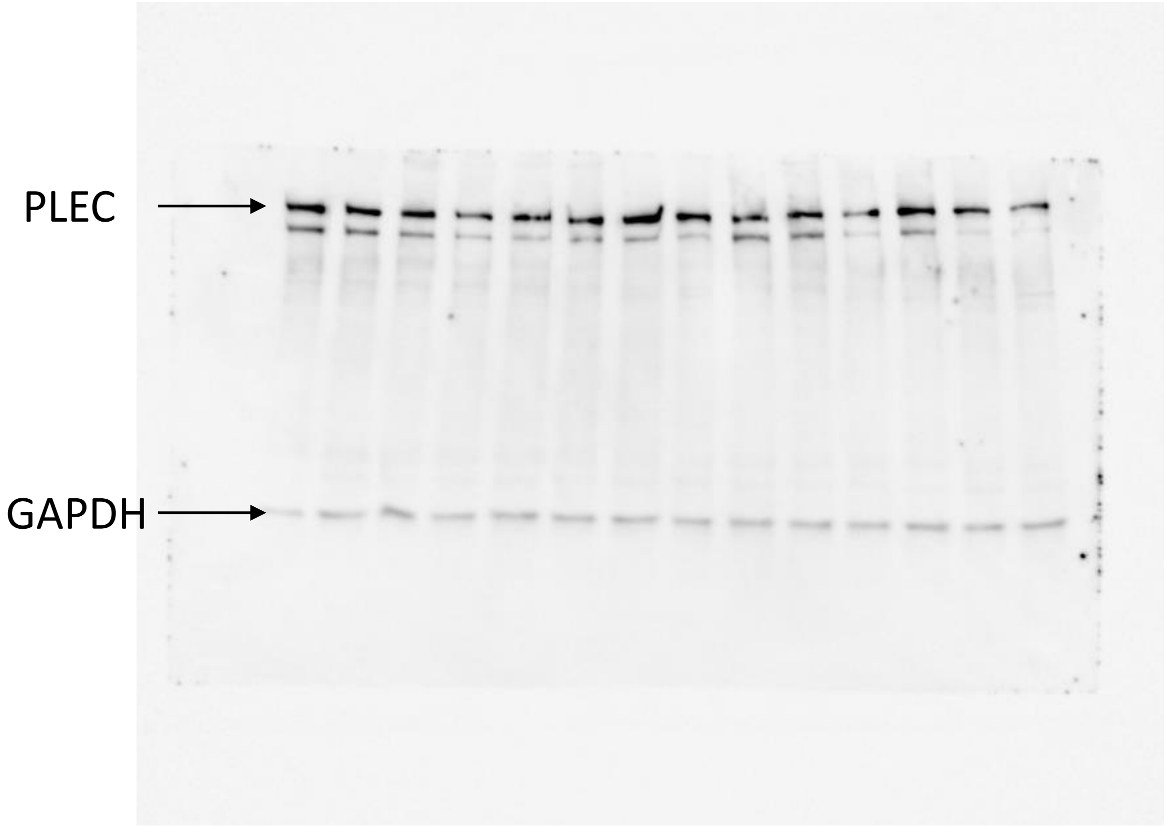

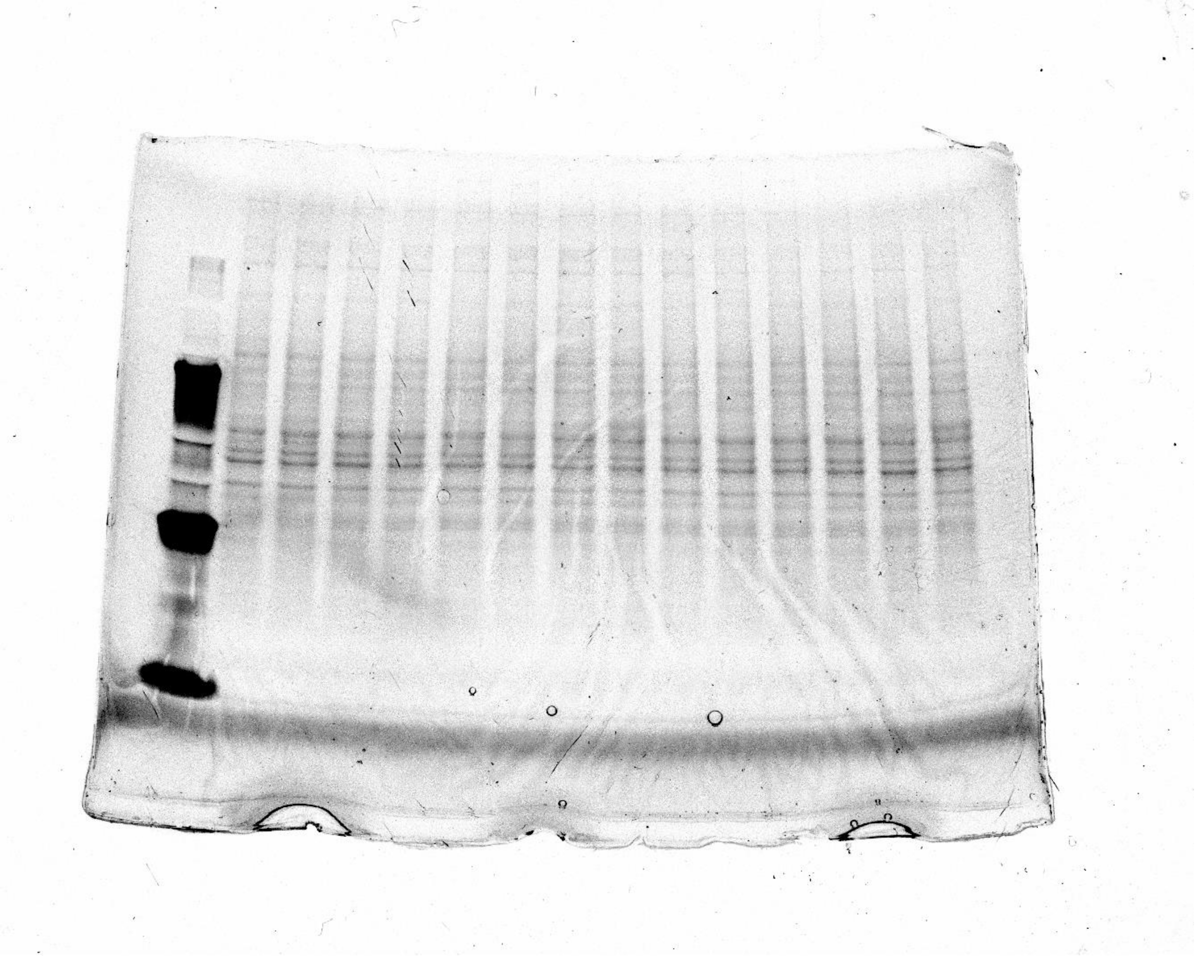

Figure 4.

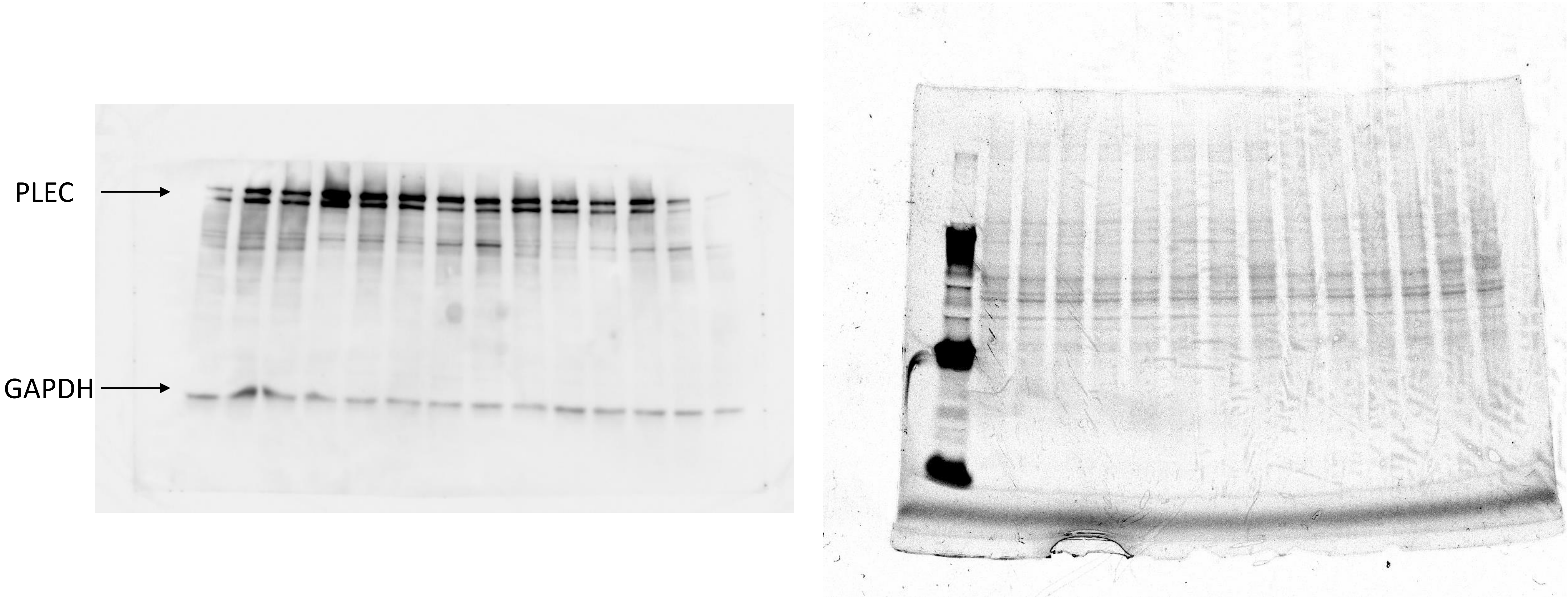

Figure S2

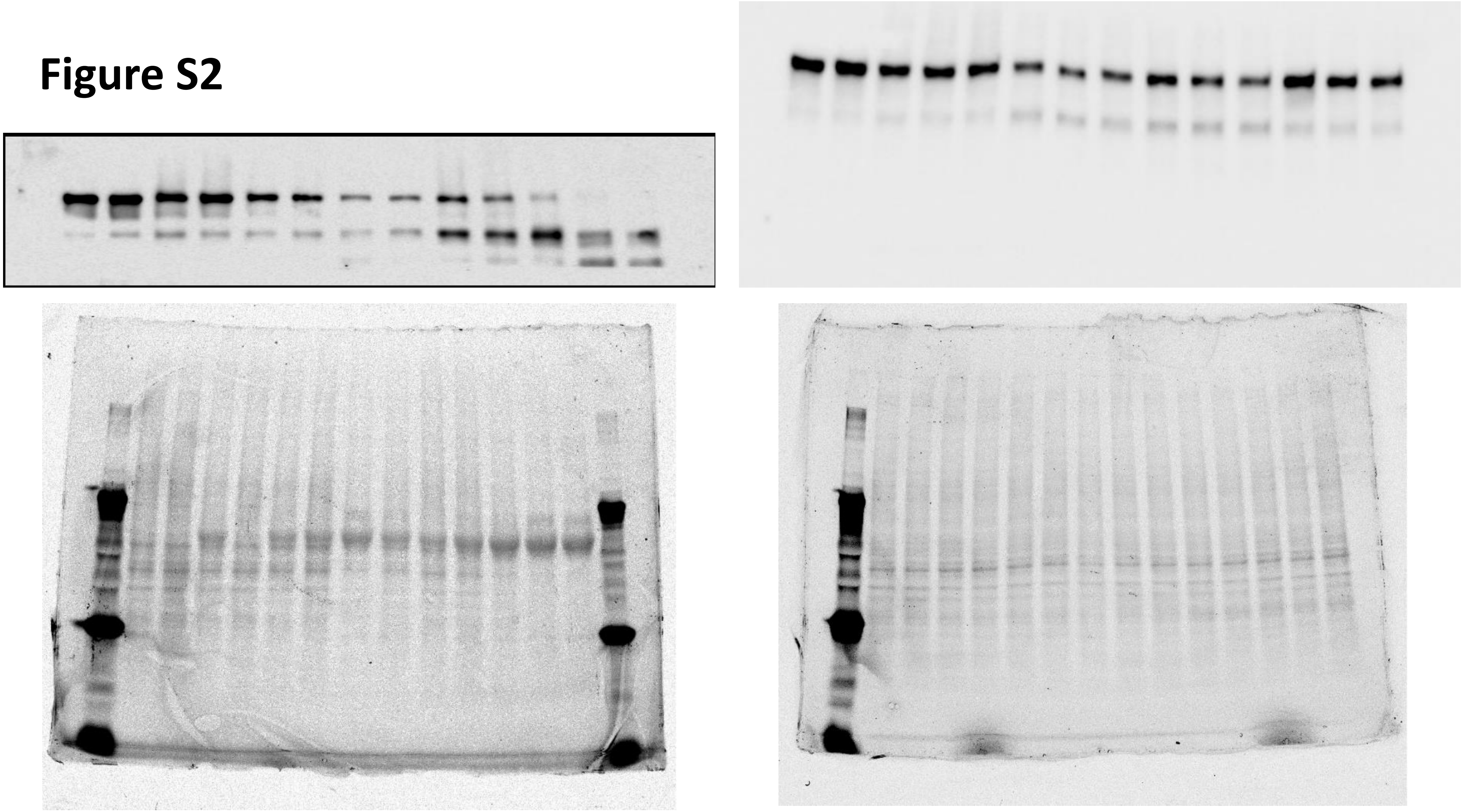

Supplement: Supplementary file 2 — Supplementary Figures. [file 41598_2022_14019_MOESM2_ESM.pdf]
